# CTD: An information-theoretic algorithm to interpret sets of metabolomic and transcriptomic perturbations in the context of graphical models

**DOI:** 10.1371/journal.pcbi.1008550

**Published:** 2021-01-29

**Authors:** Lillian R. Thistlethwaite, Varduhi Petrosyan, Xiqi Li, Marcus J. Miller, Sarah H. Elsea, Aleksandar Milosavljevic

**Affiliations:** 1 Quantitative and Computational Biosciences Program, Baylor College of Medicine, Houston, Texas, United States of America; 2 Department of Molecular and Human Genetics, Baylor College of Medicine, Houston, Texas, United States of America; 3 Department of Medical and Molecular Genetics, Indiana University School of Medicine, Indianapolis, Indiana, United States of America; University of Virginia, UNITED STATES

## Abstract

We consider the following general family of algorithmic problems that arises in transcriptomics, metabolomics and other fields: given a weighted graph G and a subset of its nodes S, find subsets of S that show significant connectedness within G. A specific solution to this problem may be defined by devising a scoring function, the Maximum Clique problem being a classic example, where S includes all nodes in G and where the score is defined by the size of the largest subset of S fully connected within G. Major practical obstacles for the plethora of algorithms addressing this type of problem include computational efficiency and, particularly for more complex scores which take edge weights into account, the computational cost of permutation testing, a statistical procedure required to obtain a bound on the p-value for a connectedness score. To address these problems, we developed CTD, “Connect the Dots”, a fast algorithm based on data compression that detects highly connected subsets within S. CTD provides information-theoretic upper bounds on p-values when S contains a small fraction of nodes in G without requiring computationally costly permutation testing. We apply the CTD algorithm to interpret multi-metabolite perturbations due to inborn errors of metabolism and multi-transcript perturbations associated with breast cancer in the context of disease-specific Gaussian Markov Random Field networks learned directly from respective molecular profiling data.

## Author summary

A frequently encountered “omic” analysis problem is to identify a subset of nodes within a weighted graph G that is both highly connected in G and belongs to S, a subset of nodes in G. For example, G may represent a biological pathway, kinetic network model, biological interaction network, or a network learned directly from data, where edges represent co-variation relationships between abundances of molecular variables. S may be the set of molecular variables that are perturbed in an individual case or in a set of disease cases relative to controls. In this work, we develop a novel information-theoretic formulation of this problem and a local search algorithm that obviate the need for computationally costly permutation testing, a statistical procedure which is typically required to establish rigorous p-value bounds for other scoring-based methods.

This is a *PLOS Computational Biology* Methods paper.

## Introduction

Weighted graphs are often used to model variability in biological systems detected from molecular profiling. Such graphs may also serve as a context for interpreting perturbations observed in independent cases. Specifically, given a co-perturbation graph G and a subset of its nodes S, corresponding to a set of variables perturbed in one or more independent cases, it is of interest to identify subsets of S that show significant connectedness within G.

While a variety of algorithms exist that address related problems, they typically do not help derive specific hypotheses by identifying specific subsets of S that are highly connected within G. Moreover, the scoring functions employed by many current algorithms typically require permutation testing to establish statistically rigorous p-values. To address both problems, we developed CTD, a novel information-theoretic algorithm that figuratively “**c**onnects **t**he **d**ots” by detecting subsets of S that are significantly connected within G, and assigns an upper bound on their p-values without the computational cost associated with permutation testing.

To demonstrate the utility of CTD, we focus on two application areas. Firstly, we apply CTD towards the diagnosis of monogenic inborn errors of metabolism using metabolomics profiling by mass spectrometry of human plasma. Mass spectrometry provides measurements of abundances of hundreds to thousands of metabolites and usage of these abundances as functional evidence of metabolic disease has already been integrated into clinical practice [1–3]. Perturbations (i.e. unusually high or low abundances) of specific metabolites observed in an individual patient are represented using z-scores comparing metabolite abundances against an established reference population, and then interpreted in the context of disease-specific networks.

Secondly, we apply CTD as an alternative to existing topology-based pathway enrichment methods in the analysis of gene expression perturbations observed in four major breast cancer subtypes (Luminal A, Luminal B, Her2 and Basal-like) from The Cancer Genome Atlas (TCGA) RNA-seq data [4,5]. RNA-seq is a powerful technology that can capture a single base pair resolution snapshot of the transcriptome. A wide variety of tools can be used to align RNA-seq reads [6,7], quantitate the number of reads per gene [8,9], and identify differentially expressed (DE) genes [10,11]. However, regardless of which pipelines are used to ultimately identify DE genes, the biological significance of the identified genes still requires interpretation. Pathway enrichment tools such as DAVID [12] are commonly used to identify pathways that are enriched for a given set of DE genes. However, current pathway analysis methods do not often clarify the relationship between DE genes and the biological mechanism driving the disease state. We address these problems by employing CTD to figuratively “**c**onnect **t**he **d**ots” between DE genes within appropriate gene co-expression networks.

## Results

### Overview of the CTD method

We consider the following general family of algorithmic problems: given a weighted graph G and a subset of its nodes S, find subsets of S that are significantly connected within G. A classic member of this family is the Maximum Clique problem, where S includes all nodes in G and where the objective is to maximize the size of the subset of S which is fully connected within G. Thus, the score employed is the size of the clique without regard to edge weights. In many “omic” applications, more complex scores are frequently applied, such as scores that take into account edge weights in G, which do not require that edges be present between all nodes within a subset to be considered strongly connected. The graph, G, may represent a biological pathway, kinetic network model, biological interaction network, or a network learned directly from data. We here focus on a class of applications where the edges of graph G represent co-variation relationships between abundances of molecular variables (e.g., metabolite abundances or gene expression levels) and S represents a set of molecular variables that are perturbed in an individual disease case or a set of cases relative to controls. One practical obstacle for more complex node set scores that take edge weights into account is the computational cost of permutation testing, which has historically been required to obtain a bound on the p-value for an extreme node set score. To address this problem, CTD provides information-theoretic upper bounds on node set p-values as an efficient alternative to permutation testing. CTD node set scores are defined by a data compression scheme, *A*, that concisely encodes subsets of S that are highly connected in G. If S can be compressed in ***I_A_***(**S**) bits and if the encoding of S by the “null” hypothesis requires ***I*_0_**(**S**) bits, by the Algorithmic Significance theorem [13], significance of the node set, S, is ***p***≤**2^−*d*^**, where *d* = ***I*_0_**(**S**)-***I_A_***(**S**). This formulation obviates the need for computationally costly permutation testing typically required to establish p-value bounds for subsets of S. Because p-value bounds are not typically tight, this entails some loss of power relative to permutation testing.

The CTD algorithm finds subsets of S with the smallest p-values by performing network walks starting from each node in S. Starting from a given “seed” node in S, the network walker is guided by diffusion of probabilities from previously encountered nodes. This search strategy is aimed at identifying any subsets of S that are highly connected in G within a short walk starting from any of the subset’s members. By the design of a compression scheme, the encoding length of S, denoted ***I_A_***(**S**) would be small when a highly connected set of nodes in S is encountered within a short network walk. Details of the method and comparisons to related methods are provided in the Materials and Methods section. In the following, we apply the algorithm to interpret multi-metabolite perturbations due to inborn errors of metabolism and multi-gene perturbations associated with breast cancer in the context of disease-specific Gaussian Markov Random Field networks learned directly from respective molecular profiling data.

### Interpretation of multi-metabolite perturbations due to inborn errors of metabolism

#### Limitations of visual interpretation in the context of biochemical pathways

**Fig 1:**
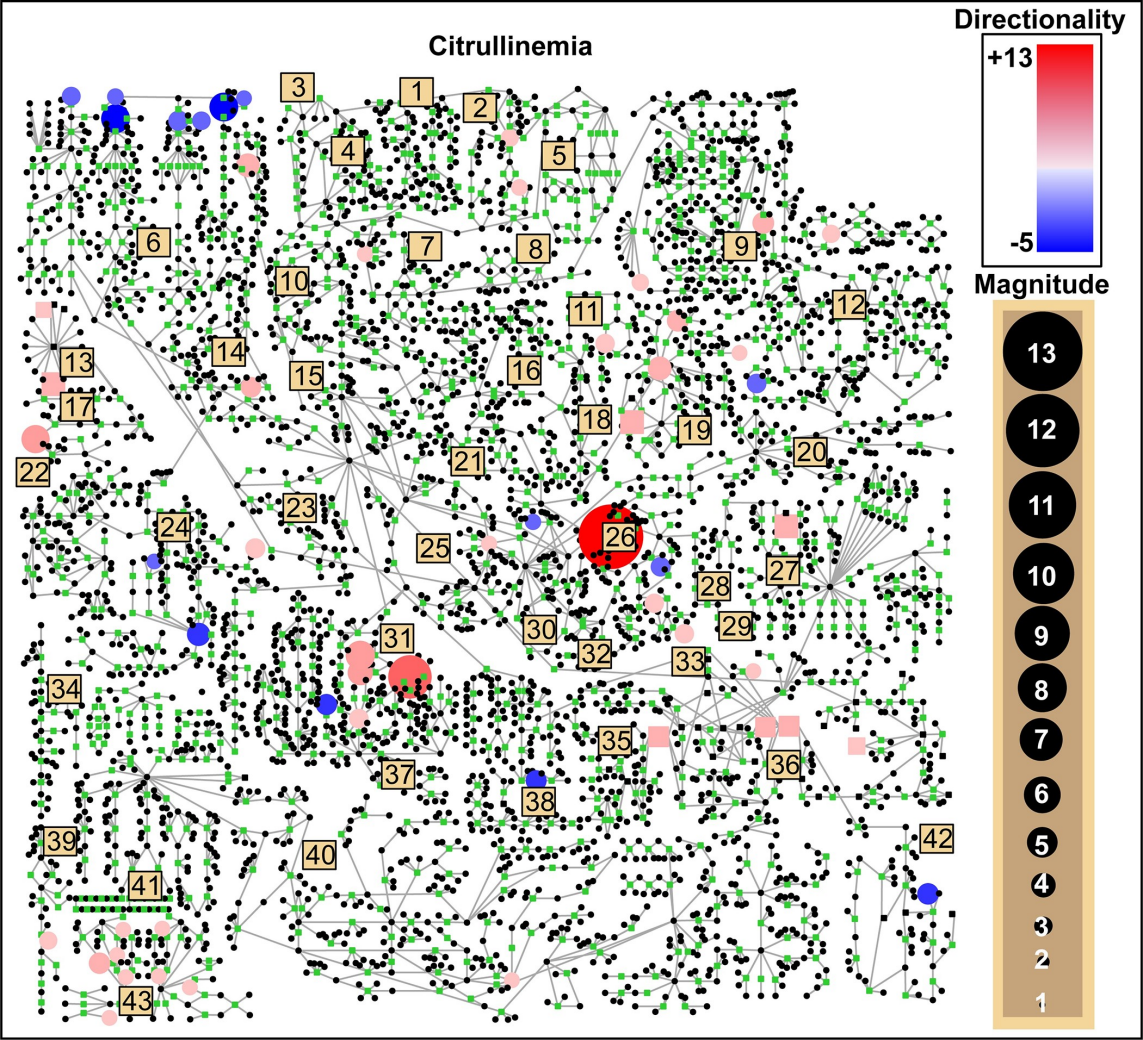
A comprehensive human metabolic pathway map with patient-specific metabolic perturbations overlaid. Representative data are shown for plasma citrullinemia patient IEM_1023. Circular nodes are metabolites and are colored red if they were perturbed upwards, and blue if perturbed downwards. The diameter of the node reflects the magnitude of the perturbation. Beige squares refer to particular sub-pathways (see **S1 Table**) which are aligned according to a coordinate space curated by Metabolon (MetaboLync Pathway Visualizations software, version 1.1.2, Copyright 2014 Metabolon, Inc., Research Triangle Park, NC, USA).

Interpretation of untargeted metabolomic profiles is currently a manual process involving visualization of perturbed metabolites in the context of known biochemical pathways. Using pathway visualization approaches, the clinician is left with the task of “connecting the dots” between perturbed metabolites within a two-dimensional map of biochemical pathways. As an example, a pathway visualization of a patient with citrullinemia is shown in **Fig 1**. Metabolite perturbations with z-scores greater than +2.0 and less than -2.0 are overlaid, and after a visual scan of these perturbations, the patient with citrullinemia shows abnormal activity in pathways such as the branched-chain amino acid metabolism (sub-pathway 6), the urea cycle (sub-pathway 26), and phenylalanine-tyrosine metabolism (sub-pathway 31).

**Fig 2:**
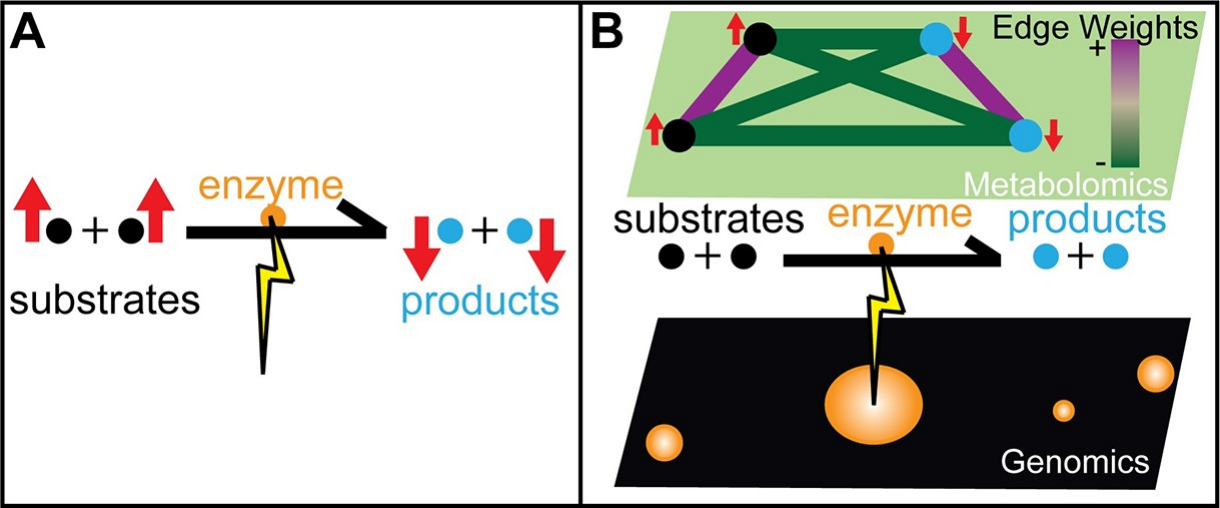
Latent covariance structure in the metabolome is induced by genetic perturbations of an enzyme. (A) Pathway visualization can unveil bottlenecks in metabolite flow when enzymes fail to function due to biallelic pathogenic mutations. Substrates are perturbed upwards and products are perturbed downwards around an affected enzyme. (B) Gaussian Markov Random Field (GMRF) networks model covariation relationships between metabolite perturbations, where each edge indicates a non-zero partial correlation between two given metabolites. When an enzyme’s function is deficient, substrate levels are perturbed upwards, product levels are perturbed downwards. This latent covariance structure within the GMRF model is characterized by positive covariance edges between substrates and between products and negative covariance edges between substrates and products.

The process of visually “connecting the dots” is both qualitative and subjective, as it depends on the specific two-dimensional layout, making some disease signatures more visually conspicuous than others. Moreover, while there is a multitude of pathway knowledgebases [14–17], they collectively capture only a fraction of metabolites that can be detected using untargeted metabolomics platforms. Even more importantly, because they reflect mechanisms identified in normal, well-functioning metabolism, these pathway knowledgebases do not capture quantitative information about disorder-specific co-variation of metabolites that may include both known substrates and products (**Fig 2**) as well as couplings that have not been previously discovered.

While pathway knowledgebases are often generated based on peer-reviewed studies of biochemistry, they often lack provenance information [18], uniform curation standards, and interoperability [19] and are prone to publication biases [20]. Metabolic pathway maps also do not characterize a multitude of metabolites detectable by mass spectrometry. Moreover, the use of different metabolite identifiers across pathway knowledgebases makes it difficult to determine if the variable profiled in a patient’s sample corresponds to a node in a pathway of interest. In our experience, a typical untargeted metabolomics profile characterizes 600–900 metabolites, and only about 33% of these can actually be reliably mapped onto pathway maps such as the one illustrated in **Fig 1**.

#### Interpretation of multi-metabolite perturbations using CTD and data-derived network models

To address problems associated with pathway knowledgebases, we decided to use data-derived partial correlation-based network models. Such networks model the strength of co-perturbation between the variety of metabolites measured in untargeted metabolomics studies. Because differences in perturbation signatures observed between disease cases and controls are encoded in the network structure as strong edge weights between disease-relevant metabolite nodes, such networks are a natural input for the CTD algorithm.

In this work, we evaluated CTD on latent covariance structures induced by genetic variation in specific enzymes (**Fig 2**). To ensure that latent covariance structures are discriminative for specific diseases, we constructed 5 disease-specific networks using metabolomics profiles from Miller et al. (2015). Using CTD, we scored sets of metabolite perturbations observed in individual patient profiles against each disease-specific network, expecting that the most significant matches would occur between patient-specific metabolite perturbation sets and networks specific to their clinical diagnosis.

**Fig 3:**
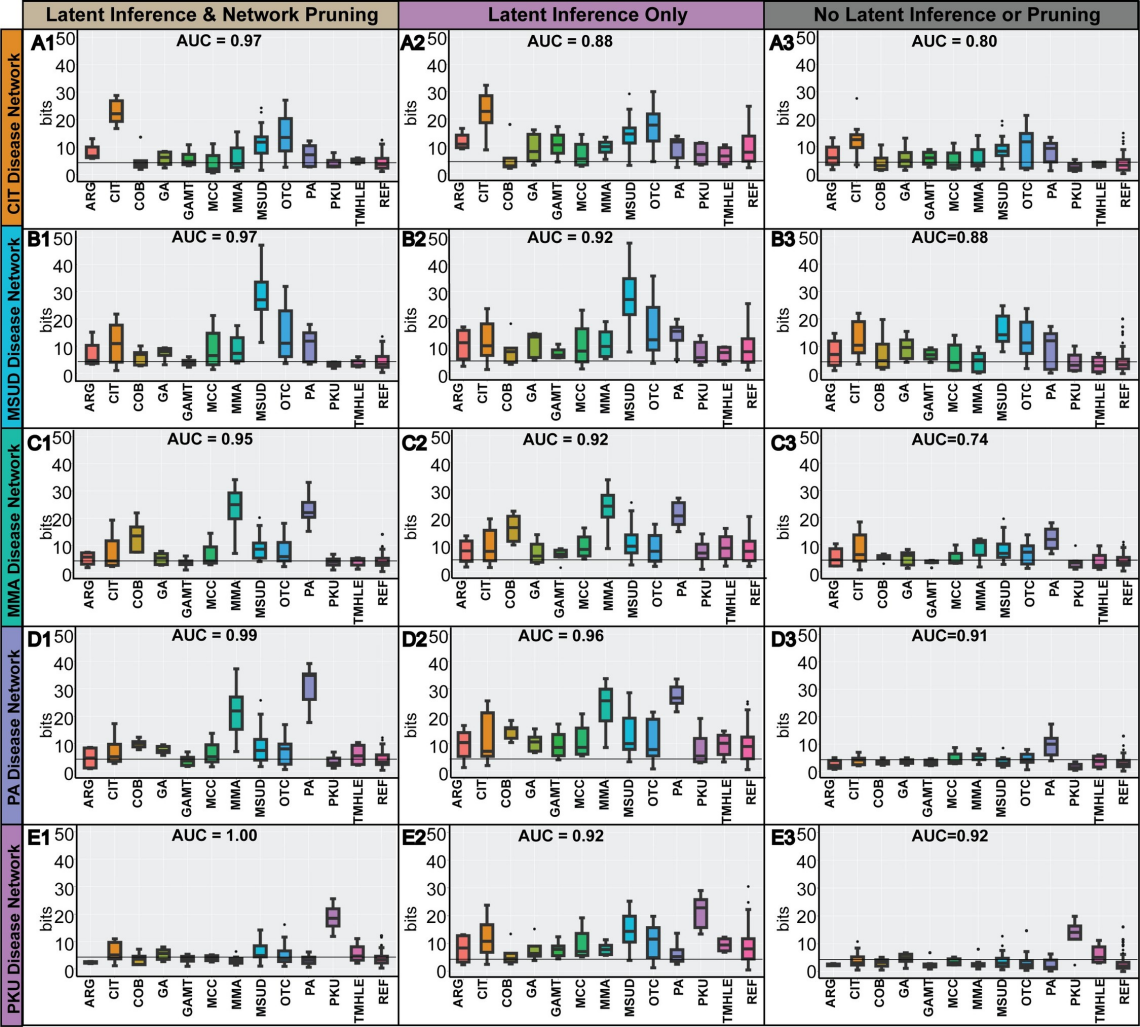
Significance of multi-metabolite perturbation signatures assigned by CTD. Using either i) discriminative latent structure inference with network pruning, ii) discriminative latent structure inference without network pruning, or iii) neither discriminative latent structure inference or network pruning during network learning, patients were scored using CTD and leave-one out cross validation against five different disease-specific network contexts: (A) citrullinemia, (B) maple syrup urine disease, (C) methylmalonic aciduria, (D) propionic aciduria, and (E) phenylketonuria. When using both discriminative latent structure inference and network pruning (column 1), disease patients showed strong significance when interpreted against the correct disease-specific network and little to no significance when interpreted with incorrect disease-specific networks. Without network pruning (column 2), the vast majority of patients across all diagnostic categories showed significantly connected submodules. Thus, network pruning improves network specificity. The added effect of removing discriminative latent structure inference (column 3) from network learning is lower sensitivity, as the disease patients show weaker signal in the correct disease-specific network context. The grey horizontal line is drawn at ~4.32 bits (i.e., FDR corrected p-value of 0.05). All scores are reported in bits, which are negative logarithms of p-values (see **Materials and Methods**).

To generate the data-derived network models, we applied three different network learning strategies: i) discriminative latent structure inference + network pruning, ii) discriminative latent structure inference + no network pruning, and iii) no discriminative latent structure inference or network pruning. Briefly, discriminative latent structure inference is a network construction strategy purposed to model the differences in perturbation signatures between two states (disease vs. control), and is derived by learning a “disease-control” network from data that is composed of a balanced set of disease samples and control samples (see **Materials and Methods**). Similarly, network pruning removes normal co-variation signatures by subtracting edges found in a “control-only” network from the “disease-control” network (see **Materials and Methods**). To establish the generalizability of our network models, we used leave-one out cross validation for each network model to create *k* disease-specific network folds, where *k* corresponds with the number of available disease metabolomics samples for a given inborn errors of metabolism (**S2 Table**). For brevity, we plot diagnoses with 4 or more representative patient samples in **Fig 3**, illustrating 12 of the 21 diagnostic classes included in the Miller et al. (2015) dataset. We show that discriminative latent structure inference is associated with higher model sensitivity, whereas network pruning is associated with higher model specificity. We also show the interpretability of CTD in **S1 Fig**, where for each patient of interest, metabolite perturbations identified by CTD can be inspected.

#### Analysis of CTD’s performance using simulation experiments

CTD establishes p-value bounds quickly, without the use of permutation testing. Inevitably, this design choice comes with some loss of power, and one question we had was how much power is lost using CTD’s computationally efficient approach. The CTD method for estimating upper bounds on p-values may be viewed as using a self-contained null hypothesis [21] without a sampling model (i.e., no permutation testing).

**Fig 4:**
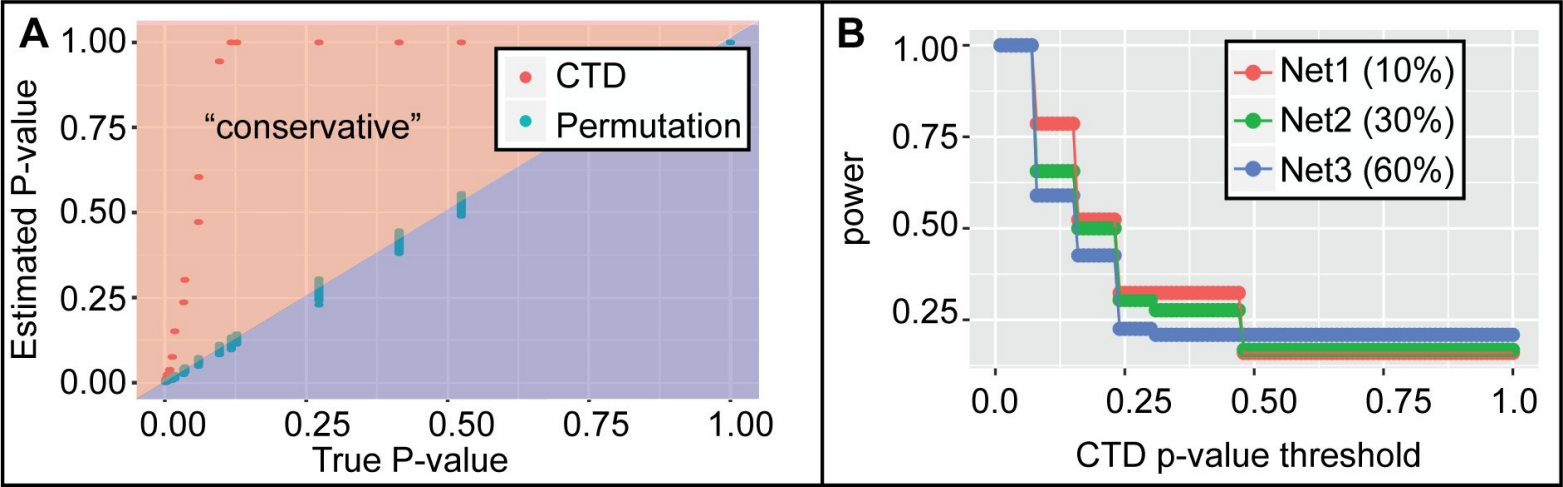
Power of indirect CTD p-value estimation compared to permutation-based p-value estimation. For three separate network structures containing 50 nodes at variable levels of connectedness (network 1: 10% connected; network 2: 30% connected; network 3: 60% connected), p-values for node sets of size 5 were estimated by three estimation methods: “ground truth” enumeration, permutation-testing and upper bound estimation by CTD. Ground truth was calculated via brute force enumeration for all (505)=2,118,760 node set outcomes of size 5. (A) CTD p-value bounds were more conservative than permutation-based p-value estimates. However, for highly connected node sets which were given more significant p-value bounds by CTD, the difference between CTD’s upper p-value bounds and the ground truth p-value is smaller. (B) Power associated with CTD upper-bounds on p-values was estimated. For all experiments where the brute force p-value was less than or equal to a given significance level (e.g., 0.05), power is calculated based on the percentage of those experiments where the CTD upper bounds p-value estimate was also less than or equal to the given significance threshold (i.e., the true positive rate). Similar to the view of the data in (A), we see that CTD’s p-value bounds show higher power for highly connected node sets compared to sparsely connected node sets.

In order to evaluate the power lost, we compared CTD’s upper bounds p-value estimation approach to permutation testing (i.e., a sampling model). We also calculated the ground truth p-value for node sets by applying brute force enumeration across ~2 million node sets of size 5 in 3 different simulated networks of size 50 with varying levels of connectedness (**Fig 4**). In **Fig 4A**, results across all three simulated networks show that CTD’s upper bounds on p-value estimates were always more conservative than p-values estimated by permutation testing and ground truth enumeration, consistent with the definition of an upper bound. Importantly, the bounds between CTD’s upper bound estimate and ground truth is tighter when the node set is highly connected in the network. We present the results for simulated networks 1–3 separately in **Fig 4B**, where we calculated the power (sensitivity) of CTD’s p-value estimates as the number of node sets where both CTD and the brute force p-value was less than a given p-value threshold, divided by the number of node sets where the brute force p-value was less than that threshold. We calculated power separately for node sets for various CTD upper-bounds p-value estimates [< = 0.01, … < = 0.50, …, < = 1.00], where the larger the threshold, the more node sets are considered. Overall, these simulations suggest that CTD is more powerful for node sets that are highly connected.

Overall, CTD’s p-value bounds approach shows less power compared to permutation testing, but the execution time gains may outweigh the power lost. Specifically, it takes an average of 0.15 seconds for CTD to estimate the upper-bounds of the p-value for a node subset of size 5 against a ~50 node graph. In contrast, it takes ~20 seconds (approximately 10 seconds to pre-compute node ranks, and 10 seconds to compute the permutations) to estimate the p-value based on 2,000 permutations. These execution times scale as the network size, subset size, and subset connectedness are increased (**Table 1**). We note that in the network of size 100 and subset size 50, there is an outlier in the timing trends listed in **Table 1**. Upon inspection, this outlier can be explained by a higher average level of connectedness in the 100 sampled node sets of size 50 in the network of size 100, compared to the other network sizes (e.g., size 500, 750 and 1,000).

**Table 1:** Execution time (in seconds) of CTD p-value bounds compared to using permutation testing for a variety of network and subset sizes. In the following timing experiments, each network was approximately 20% connected. Times for CTD’s p-value bounds estimation are reported as the average execution time observed in a sampling of 100 node sets of a given size. In the permutation testing execution times, the overhead time (in seconds) for pre-computing the node ranks is listed separately, and the additional time to perform permutation testing for one node set is denoted by a plus sign, for various subgraph sizes and network sizes.

Network Size	Overhead	k = 5		k = 10	k = 25	k = 50	k = 100
**CTD P-value Bounds Estimation**							
50	N/A	<1s		<1s	4s		
100	N/A	<1s		1s	7s	33s	
500	N/A	1s		2s	6s	14s	45s
750	N/A	2s		4s	10s	22s	64s
1,000	N/A	3s		7s	18s	40s	105s
**Permutation Testing P-value Estimation**							
50	8s		+10s	+19s	+81s		
100	75s		+10s	+18s	+58s	+269s	
500	10370s		+10s	+17s	+44s	+102s	+307s
750	25130s		+10s	+18s	+44s	+100s	+268s
1,000	53542s		+11s	+19s	+48s	+107s	+273s

#### CTD can serve as a feature selection method and as an informative covariate in Partial Least Squares regression models

In chemoinformatics, Partial Least Squares (PLS) regression modeling is the state of the art for discrimination of cases and controls. However, PLS is based on the premise that all quantitative chemical measurements included in a model are predictive of disease status, and that many samples are available to train the model. Neither of these assumptions are true for metabolomics profiling of rare inborn errors of metabolism, as it can be difficult to accumulate enough samples to properly select a set of relevant metabolites. We therefore compared CTD as a feature selection method to a basic top z-score feature selection method used in the clinic and the FSFCN algorithm [22]. For the FSFCN algorithm, we employed three different network clustering methods: InfoMap [23], Greedy Modularity Optimization (GMO) [24], and WalkTrap [25]. Metabolites selected by each of the feature selection methods are outlined in **S3 Table**. To indicate the relevance of each metabolite selected by each feature selection method, we have highlighted known biomarkers for each diagnostic category in green and novel clinically relevant metabolites not previously identified in Miller et al. (2015) in yellow. Purple-highlighted metabolites are perturbed as a result of medication or diet treatment for the disease of interest. While inclusion of just one known biomarker for each of the 5 inborn error of metabolism disorders modeled often resulted in a perfect ROC-AUC across all feature selection methods, inspecting the metabolites selected by each approach revealed interesting differences (**S3 Table**). While all feature selection methods can be modified to output a different number of selected metabolites, we chose to use an absolute value z-score threshold of 2.0 for the top z-score feature selection method approach. Similarly, for all FSFCN feature selection models, we set the threshold R–which is used during the pruning of the feature correlation network—to the 95^th^ percentile of mutual information observed between all metabolites and diagnostic class labels. Lastly, CTD selected metabolites that were in at least 50% of disease patients’ highly connected metabolite perturbation sets, where a metabolite was included as a perturbation if it corresponded to a z-score greater than 2.0 or less than -2.0. When inspecting variable importance assigned to metabolite features included in each regression model, the CTD covariate was ranked competitively amongst the selected metabolite variables (**S3 Table**), suggesting its usefulness as an informative feature for diagnosis.

### Interpretation of gene expression profiling experiments using CTD

**Fig 5:**
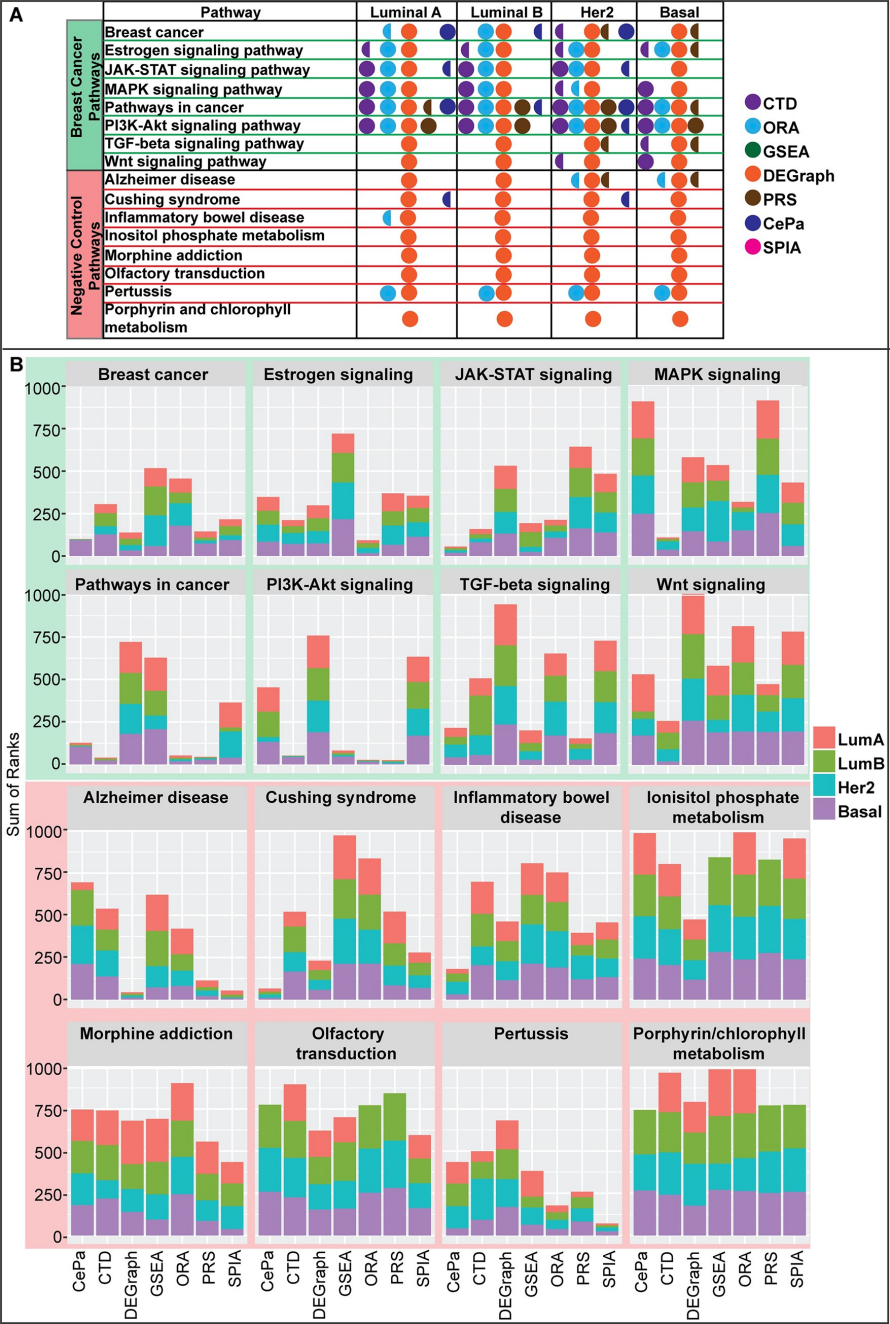
Sensitivity and specificity of pathway enrichment methods compared to CTD. Pathway ranks outputted by 7 pathway enrichment methods (CTD, ORA, GSEA [27], DEGraph [30], CePa [28], PRS [33], SPIA [29]) associated with eight breast cancer-related (highlighted in green) and eight pathways unrelated to breast cancer (highlighted in red). (A) Full circles denote a given pathway enrichment method outputted an FDR p-value < 0.05 for a given pathway. Semi-circles denote borderline significance (0.05 < FDR < 0.15). DEGraph appears to be the most sensitive pathway enrichment analysis method, followed by CTD and ORA. However, DEGraph and ORA lack specificity compared to CTD, in that they also call several pathways unrelated to breast cancer significant. (B) Stacked barplots show the pathway ranks for 8 breast cancer relevant and 8 negative control biological pathways outputted by 7 different topological- or set-based pathway enrichment methods. The stacked barplots can be interpreted by looking at two different features: overall height and the differences in height of subtype rankings. Overall height indicates that a given pathway enrichment method ranked that pathway as less important compared to competing pathway enrichment methods. Differences in subtype height within a given method reveals whether or not subtype-specific differences were captured by that particular pathway enrichment method for that pathway. Plotting the pathway ranks of breast cancer relevant pathways across methods identifies DEGraph as a poorly specific enrichment method. In contrast, CTD shows both high specificity and sensitivity.

We compared the performance of CTD against widely adopted pathway enrichment methods using gene expression profiling data of breast cancer samples generated by the TCGA Research Network [5] as a benchmark. We focused on RNA-seq profiles of breast cancer specific to the four main breast cancer subtypes (Luminal A, Luminal B, Her2, Basal-like). Specifically, we compared the p-values and ranks of eight breast cancer-relevant pathways previously reported in the literature [26] and eight negative control pathways outputted by two set-based and five topology-based pathway enrichment methods (**Fig 5**).

Two popular set-based pathway enrichment methods are over-representation analysis (ORA) and gene-set enrichment analysis (GSEA) [27]. ORA tends to over-inflate power, which results in poorer specificity, primarily due to its use of gene sampling while knowingly violating the assumption of independence between gene variables [21]. In contrast, the GSEA algorithm is often underpowered. Our use of both of these methods in both the analysis of metabolomics and transcriptomics datasets have led to similar conclusions (**S4 and S5 Tables, Fig 5**).

Topology-based enrichment methods are built around the goal of identifying features of a pathway topology that, when perturbed, will drastically impact the ability of that pathway to function normally. Like CTD, these methods use information embedded in network structures to quantify the significance of a set of nodes in the network structure. CTD assigns significance to a subset of nodes that are highly connected in a disease-specific co-perturbation network. Topology-based pathway enrichment methods assign significance in a variety of ways, focusing on features of a pathway topology that show larger opportunity for impact on normal pathway functioning, such as node centrality (CePa: [28]), or node hierarchy (SPIA: [29]). Multivariate topology-based pathway enrichment methods (DEGraph: [30]) take it one step further, by looking at features describing a node set as a whole in the context of a pathway topology, instead of looking at each member of the gene set individually and generating an aggregate score.

In order to compare the value of results outputted by GSEA, ORA and several topology-based methods (e.g., PRS, CePA, SPIA, DEGraph) to CTD, we compared both the p-values (**Fig 5A**) and pathway ranks (**Fig 5B**) of eight positive control (breast cancer-relevant) and eight negative control (unrelated to breast cancer) pathways out of 295 total KEGG pathways. When ties were observed, pathway ranks were determined by the alphabetical ordering of the pathway name. Consistent with reports from Ihnatova et al. [31] and Braun & Shah [32], we find that multivariate methods are much more sensitive than univariate topology-based enrichment methods. Univariate topology-based methods CePa [28], PRS [33] and SPIA [29] show less power to find significant pathways compared to multivariate pathway enrichment methods; however, PRS’s and CePa’s specificity is well noted (**Fig 5A**).

Previously DEGraph was reported as the most specific of several multivariate topology-based pathway enrichment methods [31], and that all methods in this class are highly impacted by sample size, an observation our experience seems to confirm. For example, even when selecting only ~50 samples, DEGraph still called 273/295 KEGG pathways significantly affected, and with the full dataset, DEGraph called 292/295 KEGG pathways significantly affected for the Luminal A breast cancer subtype, a trend which replicated across all other breast cancer subtypes. This observation illustrates that while DEGraph was the most specific multivariate topology-based method in the analysis performed by Ihnatova et al. [31], its behavior is still quite non-specific. Of the methods compared, the ranking of p-values for DEGraph was less informative for performance due to the number of ties observed, where the vast majority of p-values were approximately zero. Of note, DEGraph oftentimes identifies and assigns significance to multiple subnetworks within a given pathway. To achieve an overall pathway significance value, we combined p-values associated with multiple subnetworks using Fisher’s combined p-value, though other aggregation methods (e.g., MIN, MAX) still outputted similar results relating to the number of pathways found significant.

In summary, our results suggest that CTD strikes a unique balance between sensitivity and specificity for identifying biologically relevant pathways affected by differentially expressed genes. In all breast cancer subtype analyses, CTD ranked several breast-cancer relevant pathways (MAPK signaling pathway, Pathways in cancer, and the Wnt signaling pathway) with the most relevance across all pathway enrichment methods tested (**Fig 5B**), and avoided calling negative control pathways significant (**Fig 5A**). Moreover, CTD called the Wnt signaling pathway significantly affected for the Basal and Her2 subtypes, but called it insignificant for Luminal A and B subtypes, consistent with mechanistic differences between the breast cancer subtypes. Similarly, CTD ranked the TGF-beta signaling pathway high only for the Basal subtype. CTD also showed stronger sensitivity than GSEA and all univariate topology-based methods (i.e., PRS, CePA, SPIA) (**Fig 5A**), and showed higher specificity than DEGraph and ORA, calling fewer KEGG Pathways significant overall.

## Discussion

In this work, we developed CTD, a novel algorithm which discovers patterns of connectedness in weighted graphs. One motivation for the development of CTD was to interpret sets of molecular perturbations observed in individual cases. A key drawback observed in the majority of competing methods is their reliance upon case-control study designs which assume a multiplicity of cases and controls. In general, this limits the use of these methods to interpreting large molecular datasets, making them less useful in the diagnosis of individual cases and the study of rare genetic disorders. By pursuing an information-theoretic strategy which uses a self-contained null hypothesis, CTD overcomes this limitation and facilitates the interpretation of perturbation signatures observed in single cases. However, one notable limitation of CTD is that its p-value bounds are valid only when the size of the node subset under consideration is small in comparison to the number of nodes in the graph.

CTD was motivated by previous multivariate biomarker selection methods, where variable sets (“modules”) are in principle more informative than single perturbed variables, more statistically powerful, and are generally more reliable biomarkers for disease and treatment effects [20,34,35]. In this work, we show that CTD can leverage information inherent in data-derived co-perturbation networks to select sets of relevant perturbed variables which are highly connected in those networks.

Lastly, our work builds upon rich literature describing knowledge discovery in network structures, which has historically been devoted to mining and interpreting differentially expressed (DE) gene sets. Active module detection methods [19] in particular overlay information from molecular profiling data (e.g., gene expression) onto network structures, and leverage topological information in the network to interpret the set of DE genes. A subclass of active module detection methods applied in particular to pathway knowledgebases are topology-based pathway enrichment methods, which we also compare to commonly used set-based pathway enrichment methods as a benchmark. Our analysis confirmed the higher sensitivity associated with topology-based pathway enrichment methods previously reported in several articles [31,32]. When we compared CTD’s ability to interpret perturbation signatures observed in a large RNAseq dataset to 6 other pathway enrichment methods, CTD showed the strongest balance between sensitivity and specificity compared to all other methods tested.

In specific regards to multivariate topology-based pathway enrichment methods such as DEGraph, CTD provided higher specificity. From the perspective of a scientist aiming to identify a manageable list of mechanistic hypotheses, the lack of specificity observed is not ideal. Because DEGraph is based on Hotelling’s T^2^, we believe its lack of specificity is driven by the amount of data, in that more data allows Hotellings T^2^ to find significant differences, albeit differences of smaller effect.

Building on these previous approaches, CTD advances the state of the art by providing a generic computational method that assigns information-theoretic p-value upper bounds to perturbed variable sets in the context of disease-specific networks without incurring the often limiting computational cost associated with permutation testing. Because it relies solely on combinatorial information and does not require precise quantitation, CTD has the potential to integrate data across platforms. This is particularly relevant for metabolomics where quantitative assays are only available for a fraction of metabolites detected by untargeted assays. While pathways represent accumulated knowledge by the community, CTD can also utilize data-derived network structures which cover a much larger proportion of measurements and captures the covariance structure between variables. In the application of CTD as a pathway enrichment method, known pathways can be used to identify variables for data-derived network construction, integrating prior knowledge with data-driven information. As a whole, the unique combination of features and applications facilitated by CTD opens numerous possibilities for improved diagnosis and discovery.

## Materials and methods

### Calculation of p-values by the algorithmic significance method

Our proposed solution to the “Connect the Dots” (CTD) problem is defined by minimizing a score that is based on data compression scheme *A* which concisely encodes subsets of S that are highly connected in G. Because of close correspondence between the design of data compression schemes and probability distribution modeling, one can think of the definition of a data compression scheme as being analogous to the construction of a generative model. Along these lines, following [13], we first define P_0_ to be null and P_*A*_ to be alternative probability distributions over subsets of nodes of size k in graph G. The Algorithmic Significance theorem [13] assigns an upper bound to the p-value for any node subset S by the following formula:

$$P_{0}\left(\frac{P_{A}(S)}{P_{0}(S)}>d\right)<2^{-d}$$ (1)

Denoting the encoding length by the null distribution *I*_0_(*S*) = −*log*_2_(*P*_0_(S)) and the encoding length by the alternative hypothesis *I_A_*(*S*) = −*log*_2_(*P_A_*(S)) we rewrite (1) as follows:

$$P_{0}(I_{0}-I_{A}>d)<2^{-d}$$ (2)

Considering all subsets S of nodes of size k in a graph G with N nodes, an optimal uniform (i.e., “null”) data compression scheme would encode each subset in I0(S)=log2((Nk)) bits. For small subsets (k<< N) this amounts to fixed-length codes of about log_2_(N) bits per node. To refute this “null” hypothesis, we define an alternative data compression scheme *A* that uses G to compress S in *I_A_*(S) bits.

### CTD data compression scheme and algorithm

**Fig 6:**
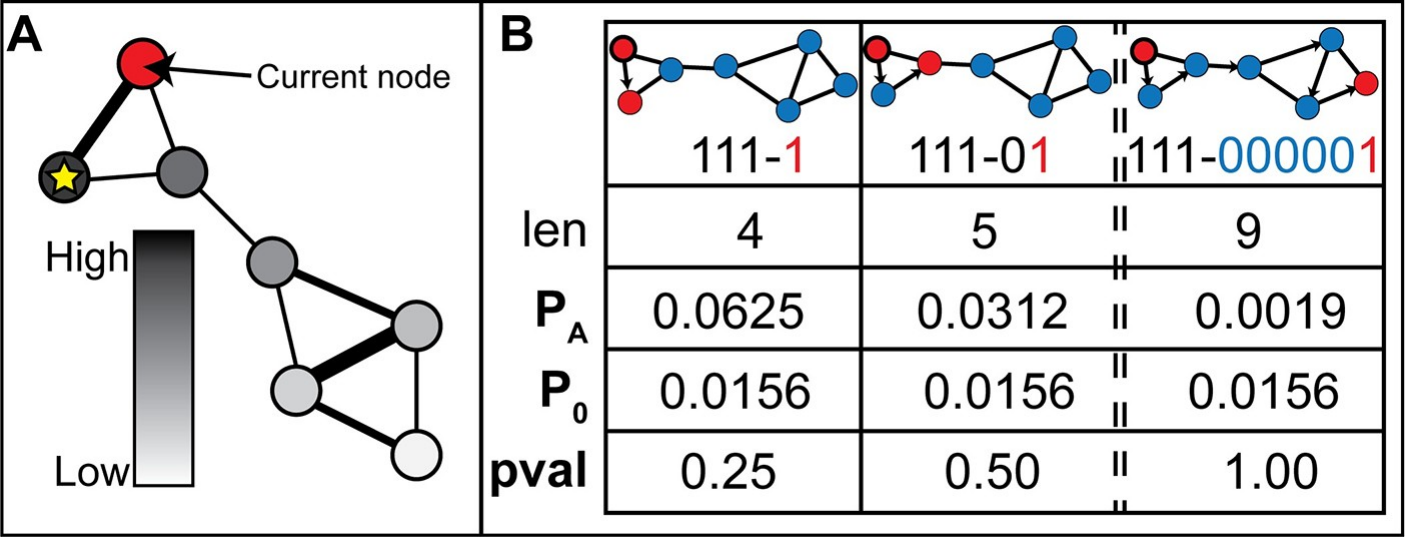
Diffusion of probability from a starting node and a diffusion-based encoding algorithm. (A) Node color denotes a gradient of probability inherited from the diffusion of probability from the current node (in red). The node with the yellow star denotes the node which inherited the highest probability. (B) A set is assigned higher probability (P_A_) if it is highly connected and assigned lower probability if it is sparsely connected in the graph. The arrows point to the node the diffusion-based node ranking algorithm ranked highest, based on diffusion of probability from the previous encoded node. While the p-value of the most connected node set of size 2 for the graph illustrated here is 0.25, with larger graphs and larger subset sizes, the potential for further compression increases, and thus, more significant p-values are possible.

The compression scheme A consists of an encoding and decoding algorithm that take advantage of the fact that at least some of the nodes in S are “close neighbors” in G and can thus be encoded concisely (I_A_(S) is small) without loss of information. In the following, we assume that the size of S is much smaller than the number of nodes in G. The encoding algorithm starts by encoding one of the nodes in S using about log_2_(N) bits. From this starting (“seed”) node, a network walker determines its next step by diffusing probability scores along the graph edges, diffusing proportionally by edge weight, from the seed node (**Fig 6A** and **Table 2**). The node inheriting the largest probability from the seed node defines the next step in the path of the network walker and this node gets added to the list of node rankings determined from a given seed node.

**Table 2:** A probability diffusion algorithm. A probability score is propagated through a network structure starting from an initial starting node (sn). In lines 4–13, probability is split preferentially between unvisited network neighbors of the starting node by edge weight and propagated recursively to secondary neighbors until the probability being diffused is less than a defined parameter, thresholdDiff (default set to 0.01). If the starting node, sn, has no unvisited neighbors, p1 is distributed uniformly amongst all unvisited nodes, regardless of proximity to sn (lines 15–16).

A Probability Diffusion Algorithm
**input:** p1, thresholdDiff, sn, G, vN, adj_mat p1 [float]–probability to be divided across network nodes thresholdDiff [float]–probability threshold at which diffusion truncates sn [string]–the node name of an initial starting node G [hash]–node names are KEYS, node probabilities are VALUES vN [vector]–visited nodes, a subset of node names (KEYS) in G adj_mat [matrix]–the weighted adjacency matrix of the network
**output:** G G [hash]–node names are KEYS, node probabilities are VALUES DIFFUSE_PROB (p1, thresholdDiff, sn, G, vN, adj_mat) 1 *UNsn = unvisited neighbors of sn* 2 *UNsnEw = edge weights between sn and each UNsn* 3 **if** *UNsn ≠ ∅* **then** 4 *sum_ewghts = sum (UNsnEw)* 5 **for each** *UNsn[i]* **in** *UNsn* **do** 6 inherited_prob = p1 * (UNsnEw[i] / sum_ewghts) 7 *G[UNsn[i]] = G[UNsn[i]] + inherited_prob* 8 **if** *inherited_prob / 2 > thresholdDiff* **then** 9 *G[UNsn[i]] = G[UNsn[i]]–inherited_prob / 2* 10 *vN*.*push (UNsn[i])* 11 *G = DIFFUSE_PROB (inherited_prob / 2*, *thresholdDiff*, *UNsn[i]*, *G*, *vN*, *adj_mat)* 12 **end if** 13 **end for** 14 **else** 15 *u_vN = KEYS (G) –vN* 16 *G[u_vN] = G[u_vN] + p1 / length (u_vN)* 17 **end if** 18 **return** *G*

The ranking of all subsequent nodes is determined by diffusion from the most recently encoded node (**Table 3**). Going down the ranked list, the encoding algorithm communicates whether or not the highest ranked node is in S by a single bit (“1” = yes; “0” = no), creating a bitstring, B (**Fig 6B**). After a run of “0”-s in B reaches a preset length threshold (“num_misses”), suggesting that there are no more “close neighbors” left in S, the algorithm reverts to encoding the remaining nodes in S using about log_2_(N) bits per node (i.e., fixed-length codes). The larger the number, F, of “close neighbor” nodes from S that are found (“F” standing for “found”) and encoded within B, the larger the compression and, consequently, the smaller the p-value (**Fig 6B**). The encoding process is attempted from each node in S and the encoding from a given seed node that minimizes the encoding length is selected for computing the final encoding length. The code produced consists of the following three blocks: (i) the encoding of the first node using about log_2_(N) bits; (ii) the bitstring, B, of length |B|, which encodes the F nodes found among the “close neighbors”; and (iii) the direct encoding of the remaining nodes using about log_2_(N) bits for each node. The total encoding length is calculated as follows:

$$I_{A}=(|S|-F)*{log}_{2}(N)+|B|$$ (3)

**Table 3:** A diffusion-based node ranking algorithm. A network walker uses a probability diffusion algorithm described in Table 2 to decide which nodes to visit in a network. Starting from a given node, *snext*, the network walker’s steps are recorded in *vns* (“visited nodes”). We record the network walker’s visited nodes, *vns*, for each starting node in S in a hash object, noderanks.

A Diffusion-Based Node Ranking Algorithm
**input:** G, S, num_misses, p1, thresholdDiff, adj_mat G [hash]–node names are KEYS, node probabilities are VALUES S [vector]–a subset of node names (KEYS) in G num_misses [int]–number of consecutive misses that terminates the walk p1 [float]–probability to be divided across network nodes thresholdDiff [float]–probability threshold at which diffusion truncates adj_mat [matrix]–the weighted adjacency matrix of the network
**output:** noderanks noderanks [hash]–node names in S are KEYS, vectors of node names visited are VALUES SINGLE_NODE_DIFFUSION (G, S, num_misses, p1, thresholdDiff, adj_mat) 1 *noderanks = Hash()* 2 *vns = []* 3 **for each** *node* **in** *S* **do** 4 *vns*.*push (node)* 5 *n_miss = 0* 6 *snext = node* 7 **while** (**not all** *S* **in** *vns*) **&** (*n_miss* < *num_misses*) **do** 8 *G = DIFFUSE_PROB (p1*, *thresholdDiff*, *snext*, *G*, *vns*, *adj_mat)* 9 *snext = KEYS (which*.*max(G))* 10 **if** *snext* **in** *S* **then** 11 *n_miss = 0* 12 **else** 13 *n_miss + = 1* 14 **end if** 15 *vns*.*push (snext)* 16 **end while** 17 *noderanks[node] = vns* 18 **end for** 19 **return** *noderanks*

Several options are provided to achieve appropriate trade-offs between computational efficiency and performance. Those trade-offs involve the diffusion step and the pre-computing of node ranks versus one-off computation. First, the diffusion algorithm terminates when the probability to be diffused reaches a small value (the ‘thresholdDiff’ parameter, default is 0.01). This step may not affect the ranking of very close nodes but may affect the ranking of more distal nodes. Second, particularly when a large number of subsets need to be encoded, pre-computing node ranks allows for much faster computation compared to one-off computation of node subsets.

While we have described a diffusion-based encoding algorithm here, in fact only the encoding length of the binary code outputted from the encoding algorithm is important for our purposes. To convert the encoding lengths into probabilities using the Kraft-McMillan Inequality, we also need to demonstrate that the code is uniquely decodable. The following two-stage decoding algorithm may be constructed: the first stage takes the string of bits representing the three concatenated blocks of bits and identifies boundaries between them, the boundary of the first block being detectable since its length is fixed to log_2_(N) bits and the boundary of the second block being detectable because the ‘num_misses’ parameter is set to a fixed value shared between the encoder and decoder before transmission and does not enter into the calculation of the encoding length; the second stage parses the first and third blocks using a shared node codebook and parses the second block using the shared weighted graph and by mirroring the steps of the encoding algorithm to identify the nodes encoded by the bitstring, B. Note that this encoding scheme also ensures that the encodings acceptable by the decoder are unique. The uniqueness follows from the observation that if even only one bit is changed in the first or third blocks, the decoding algorithm will fail to decode the encoding of the original subset, S, because the modified fixed length bitstrings will point to different nodes in the shared codebook. Similarly, if just one bit is changed in the second block, the decoder will also fail to decode the encoding of the original subset, S, as every bit in the network walker’s path denotes membership (“1”) or non-membership (“0”) in S. Therefore, the encoding of S is unique.

### Comparison to other network methods

In general, as outlined in Mitra et al. [19], CTD may be described as performing active module detection on differential networks, and may be broadly categorized as an information propagation-based search algorithm. It is important to distinguish CTD from existing network clustering, network motif detection, and active module detection methods in the literature. The network clustering method InfoMap [23] is a particularly close comparison as it also uses information theory and data compression principles to identify important substructures within a network. The primary difference between CTD and network clustering methods as a whole lies within the question and scope of the problem they solve. InfoMap and other network clustering and community detection methods are concerned with digesting an entire network structure and partitioning the network structure into communities. Of note, there are no significance values associated with the subgraphs outputted by network clustering methods: these communities are a solution to an optimization problem, and the output does not aid in the interpretation of these communities. In contrast, CTD does not attempt to digest an entire network model, but instead is concerned with identifying small sets of interpretable features. Given a subset of nodes, S, within a network, CTD outputs highly connected subsets of S in a given network. In other words, CTD uses network connectivity information in disease-specific networks to “match” patient-specific perturbations with a disease state.

Network motif detection methods [36–39] also appear similar to CTD at first glance, in that they also evaluate the significance of a subgraph within a network context. The primary methodological difference between network motif detection methods and CTD, however, is that network motif detection methods assign higher significance to subgraphs which have topologies that occur at higher frequencies than expected by chance; whereas, CTD assigns significance to subgraphs which show significant connectedness. Another primary methodological difference between network motif detection methods and CTD is that CTD can assign significance to a subgraph without the use of permutation testing or enumeration. In contrast to CTD’s encoding length subgraph scoring function which performs local search, network motif subgraph scoring functions require analysis of graphs in their entirety and are often challenged with poor running times on large networks [38].

Arguably, of all classes of algorithms mentioned above, CTD is most similar to existing network-based active module detection methods, such as CePa [28], DEGraph [30], HotNet [40] and HotNet2 [41], MATISSE [42], Multi-Dendrix [43], NetWalk [44], PARADIGM [45], PRS [33], and SPIA [29]. The main methodological difference is that CTD does not need to perform permutation testing in order to arrive at p-values; whereas almost every other method in this category does (**Table 4**). Furthermore, these methods all use interaction or pathway knowledgebases as the primary network structure used to reason about subgraphs, whereas CTD uses data-derived partial correlation network structures. Moreover, because of its generic formulation, CTD can accommodate analysis in both the functional genomics and metabolomics research contexts, whereas the majority of methods in this class focus on interpreting signatures observed in transcriptomics datasets alone.

**Table 4:** Comparison of active module detection methods with CTD.

Method	Network Structure	Biological Data	Network Propagation Used?	Optimization/Subgraph Search Performed?	Module Assigned Significance?	Permutation testing?
CTD	Gaussian graphical models	Functional -omics	YES	YES	YES	NO
CePa	Pathway Knowledgebases	Gene expression	NO	NO	YES	YES
DEGraph	Pathway Knowledgebases	Gene expression	NO	YES	YES	NO
HotNet/HotNet2	Interaction Knowledgebases	Somatic mutation	YES	YES	YES	YES
MATISSE	Interaction Knowledgebases	Gene expression	NO	YES	NO	NO
Multi-Dendrix	Interaction Knowledgebases	Somatic mutation	NO	YES	YES	YES
NetWalk	Interaction Knowledgebases	Gene expression, RNAi screen	YES	NO	NO*	N/A
PARADIGM	Pathway Knowledgebases	Copy number, Gene expression, Proteomics	YES	NO	YES	YES
PRS	Pathway Knowledgebases	Gene expression	NO	NO	YES	YES
SPIA	Pathway Knowledgebases	Gene expression	NO	NO	YES	YES

* Edge probabilities are computed as Edge Flux scores. The authors state that statistical procedures on this distribution can be applied, but do not offer module significance inherently.

### Construction of co-perturbation networks

We apply the CTD method to metabolomic and transcriptomic networks learned directly from disease case and negative control profiling data. The learning of differential networks from pairwise partial correlations of variables also involves a “pruning” of edge relationships that differ between two network conditions (a disease+control network vs. a control network) which model the same metabolites. Edges present in a control network are thought to be associated with normal variation signatures (e.g., signatures due to circadian rhythms, age, gender, common medications, etc.) and are discarded in the network pruning stage. The pruned disease+control differential network, called a disease-specific network, is then used to “score” sets of perturbed (up or down) variables.

### From untargeted metabolomics profiles

We apply the CTD method to interpret multi-metabolite perturbations due to specific inborn errors of metabolism. The data included untargeted metabolomic profiles from Miller et al. (2015). We included diagnoses that were represented by at least 8 patient samples with the exception of guanidinoacetate methyltransferase and ornithine transcarbamoylase deficiency, because they were described in Miller et al. (2015) as having no disease-related biomarkers present in the data. Five disease cohorts showed rich disease signatures and were included in the analysis, including citrullinemia (n = 9), maple syrup urine disease (n = 18), methylmalonic aciduria (n = 9), propionic aciduria (n = 9) and phenylketonuria (n = 8). Included in the Miller dataset is also 68 untargeted “control” metabolomic profiles from presumably healthy individuals. For each patient sample, untargeted metabolomics profiling revealed ~600–900 metabolites. It is important to note that though there are a small number of profiles per disease cohort, each of these profiles were z-scored against a rich reference population comprised of all 186 patient samples included in the Miller dataset. Thus, perturbation z-scores contained in just one profile of a disorder contains more information than can be gathered from a single individual.

**Fig 7:**
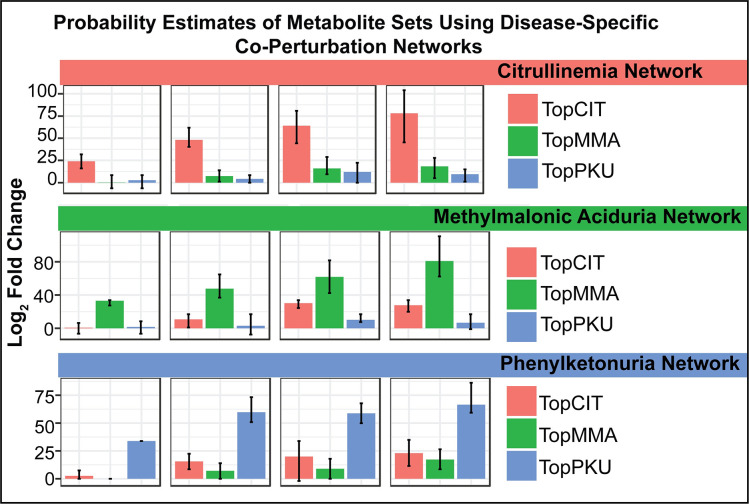
The probability of a metabolite set depends on the disease-specific network used in the encoding process. The probability of the 5, 10, 15 or 20 most perturbed metabolites across (A) citrullinemia samples (B) methymalonic aciduria samples or (C) phenylketonuria samples is much larger when using the network learned from patients with citrullinemia, methylmalonic aciduria, and phenylketonuria, respectively, compared to the probability of the same metabolite set when using a permuted network. Error bars indicate signal observed across several network folds when using a leave-one-out cross validation scheme for network learning and scoring.

We have found that disease-specific network models, as compared to models learned from a mixture of patients with a variety of clinical phenotypes, capture disease-specific metabolite perturbations more sensitively. For example, if one learns a graph from data collected from patients with citrullinemia and negative controls and then compares the probability of the set of 5, 10, 15 or 20 metabolites most perturbed across patients diagnosed with citrullinemia, one will see a large increase in the probability assigned to the metabolite set when using the graph learned from patients with citrullinemia compared to a permuted graph (**Fig 7**). Here we define a “permuted” graph as the graph learned from negative control samples with node labels randomly permuted. Likewise, we see a similar result for the top 5, 10, 15, and 20 perturbed metabolites seen in all other diagnoses, where the probability assigned to each set was much larger when using a corresponding disease-specific graph compared to a permuted graph. For brevity, only methylmalonic aciduria and phenylketonuria are shown alongside citrullinemia in **Fig 7**.

Therefore, to make the co-perturbation networks most useful for detecting disease-specific perturbations, we constructed disease-specific “co-perturbation networks” from metabolomic profiles of diseased patients and controls, for all inborn errors of metabolism, separately (**S2 Table**). A metabolomic co-perturbation network is a weighted graph where the nodes represent metabolites and edges connect metabolites that tend to be co-perturbed in the specific inborn error of metabolism disorder. The graphs are modeled as Gaussian Markov Random Field (GMRF) networks. A GMRF network can be estimated by inverting the covariance matrix of an original dataset, outputting the original dataset’s precision matrix. When the original dataset is under-ranked (e.g., having more features than samples), the graphical lasso algorithm can be used to estimate the precision matrix. We used the graphical lasso algorithm implemented in the R package *huge* (v1.2.7), where edge weights are the estimated partial correlation between any two metabolites.

**Fig 8:**
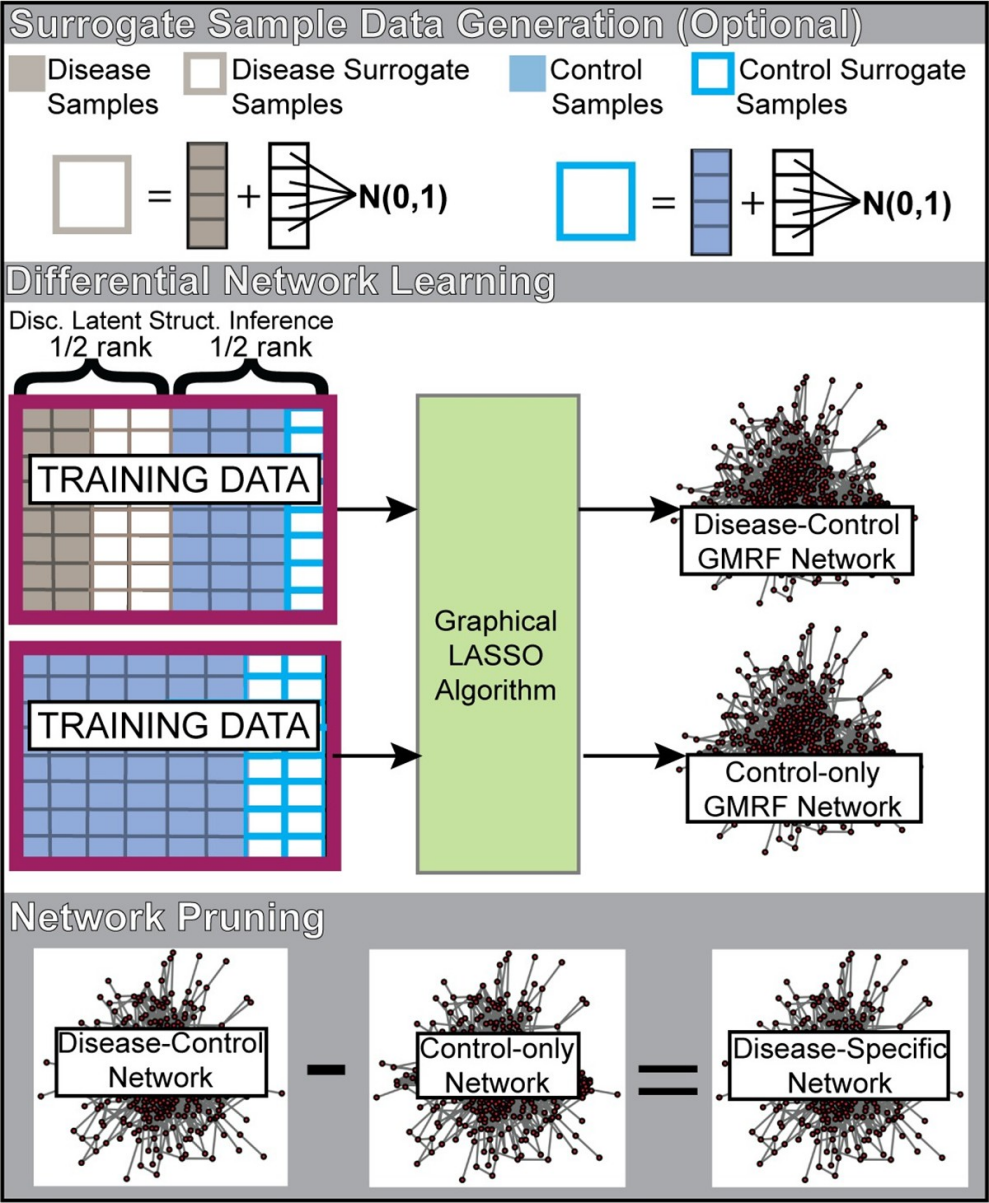
Construction of disease-specific co-perturbation networks. Two networks are learned using a Gaussian Markov Random Field network learner. The first network (“disease-control” network) is learned from disease profiles, disease surrogate profiles, control profiles, and surrogate control profiles. Importantly, half the matrix rank is composed of disease and/or disease surrogate profiles and the second half of the matrix ranks is composed of control and/or surrogate control profiles. A second network composed of only control and surrogate controls is also learned (“control-only” network). A final pruning stage subtracts edges from the disease-control network that are also found in the control-only network, outputting the disease-specific network. Disc. Latent Struct. Inference–Discriminative latent structure inference; GMRF–Gaussian Markov Random Field; N(0,1)–the standard normal distribution.

Since inborn errors of metabolism are very rare diseases with very few samples available to learn a GMRF network, we also added surrogate profiles to fill in the rank of the data matrix used for network learning (**Fig 8**). Surrogate profiles are copies of each unique disease or control sample that get added to a randomly generated vector, where each element is drawn from a standard normal distribution. Both disease and control surrogate profiles were used, where approximately half of the matrix rank was composed of disease and disease surrogate profiles and the remaining half of the matrix rank was composed of control and control surrogate profiles. Including both examples of disease and control profiles in the training data (“discriminative latent structure inference”) introduces a hidden variable representing the disease state associated with each sample, allowing the network to model the specific metabolomic differences between two conditions (disease vs. control). Lastly, we learned a “control-only” network from just the control and surrogate control profiles, which we use in the network pruning stage.

For rare disease models, where only a small number of disease cases was available for network learning, we performed leave-one-out cross validation to ensure that the resulting disease-specific networks are generalizable for data outside the data used for network learning. We compare different network learning strategies and show that the network pruning stage is important for model specificity and that discriminative latent structure inference is important for model sensitivity (**Fig 3**).

### From RNAseq profiles

We also apply the CTD method to interpret multi-gene perturbations identified in TCGA breast cancer RNA-seq data. In contrast to the metabolomics application, where we focus on sets of variables perturbed in individual cases, we here focus on differentially expressed genes observed in subtypes of breast cancer tumor samples compared to normal breast tissue samples. Level 3 (i.e., log2 transformed RSEM normalized+1 count data) processed data from TCGA breast cancer RNA-seq (IlluminaHiSeq) profiles were extracted from the Xena Browser and then normalized using DESeq2’s median of ratios method via the estimateSizeFactors() function to eliminate confounds of sequencing depth, and/or RNA composition. For the subset of genes found in a given KEGG pathway (295 in total), RNA-seq profiles associated with a particular breast cancer subtype alongside healthy control sample profiles were used to learn subtype-specific pathway co-expression networks. A control-only co-expression pathway network was also learned for use in network pruning. Differential expression analysis was performed by the limma algorithm implemented in the R package *EnrichmentBrowser* (v2.12.1) and genes with an absolute-value differential expression fold change greater than the 95th percentile across all genes were interpreted in all 295 pathway contexts in the downstream CTD pathway enrichment analysis.

## Supporting information

S1 Fig Disease-relevant metabolite features identified by CTD. Disease-relevant metabolite features shown in a network context are visualized for select patients from Miller et al. (2015) with known diagnoses. In each of the modules detected for each patient in (A)-(E), several known biomarkers associated with the patient’s true diagnosis are included, indicating that the metabolite features selected by CTD as informative to diagnosis are also clinically relevant for diagnosis. (A) Patient IEM_1017 was diagnosed with citrullinemia, and the module detected for this patient included biomarkers for citrullinemia, such as citrulline, homocitrulline, 3-ureidopropionate and uridine. (B) Patient IEM_1058 was diagnosed with maple syrup urine disease (MSUD), and the module detected for this patient included biomarkers for MSUD such as allo-isoleucine, 4-methyl-2-oxopentanoate, 3-methyl-2-oxobutyrate, leucine, isoleucine, alpha-hydroxyisovalerate, 3-hydroxyisobutyrate, isovalerylcarnitine, 2-methylbutyrylcarnitine, and hydroxyisovalerylcarnitine. (C) Patient IEM_1051 was diagnosed with methylmalonic aciduria (MMA), and the module detected for this patient included biomarkers for MMA, such as 2-methylmalonyl carnitine, propionylcarnitine, tiglyl carnitine, hydroxyisovalerylcarnitine, and 3-hydroxypropanoate. (D) Patient IEM_1093 was diagnosed with propionic aciduria (PA), and the module detected for this patient included biomarkers for PA, such as 2-methylmalonyl carnitine, propionylcarntine, 3-hydroxypropanoate, and glycine. (E) Patient IEM_1105 was diagnosed with phenylketonuria (PKU), and the module detected for this patient included biomarkers for PKU, such as phenylalanine, n-acetylphenylalanine, gamma-glutamylphenylalanine, and phenyllactate.
(TIF)

S1 Table List of pathway map sub-pathways illustrated in Fig 1. Pathway number corresponds to the number in the beige rectangles observed in Fig 1.
(XLSX)

S2 Table Description of disease-specific network folds. Gaussian Markov Random Field network model estimation was performed using the graphical lasso algorithm. Alongside cases, 68 control profiles were used in network learning. Importantly, for any given network fold, we selected metabolites that were measured in large numbers of patients in both the included control and disease profiles.
(XLSX)

S3 Table Comparison of CTD to a network-based feature selection method, and a top z-score approach used in the clinic. Green highlighted metabolites are known biomarkers for the disorder of interest. Yellow-highlighted metabolites are clinically relevant perturbations not described in Miller et al. (2015) for the given disorder. Purple-highlighted metabolites are more likely due to disease-related medication or diet changes, based on evidence from the literature or clinical inspection.
(XLSX)

S4 Table Top pathway hits using over representation analysis for select patient samples. Only pathways with a nominal p-value < 0.05 are shown.
(XLSX)

S5 Table Top pathway hits using metabolite set enrichment analysis for several inborn errors of metabolism. Only pathways with a nominal p-value < 0.05 are shown.
(XLSX)
