# Correction: CTD: An information-theoretic algorithm to interpret sets of metabolomic and transcriptomic perturbations in the context of graphical models

**DOI:** 10.1371/journal.pcbi.1009551

**Published:** 2021-10-25

**Authors:** Lillian R. Thistlethwaite, Varduhi Petrosyan, Xiqi Li, Marcus J. Miller, Sarah H. Elsea, Aleksandar Milosavljevic

There is a typo in Eq 1. A log2() is missing before the fraction $\frac{P_{A}(S)}{P_{0}(S)}$. The authors have provided the correct version of the equation below.


$$P_{0}\left(\log_{2}\left(\frac{P_{A}(S)}{P_{0}(S)}\right)>d\right)<2^{-d}$$
