# Supplementary figures and images for: CTD: An information-theoretic algorithm to interpret sets of metabolomic and transcriptomic perturbations in the context of graphical models
Source: PLoS Comput Biol. 2021 Jan 29;17(1):e1008550. doi: 10.1371/journal.pcbi.1008550 (PMC7875364; doi:10.1371/journal.pcbi.1008550)

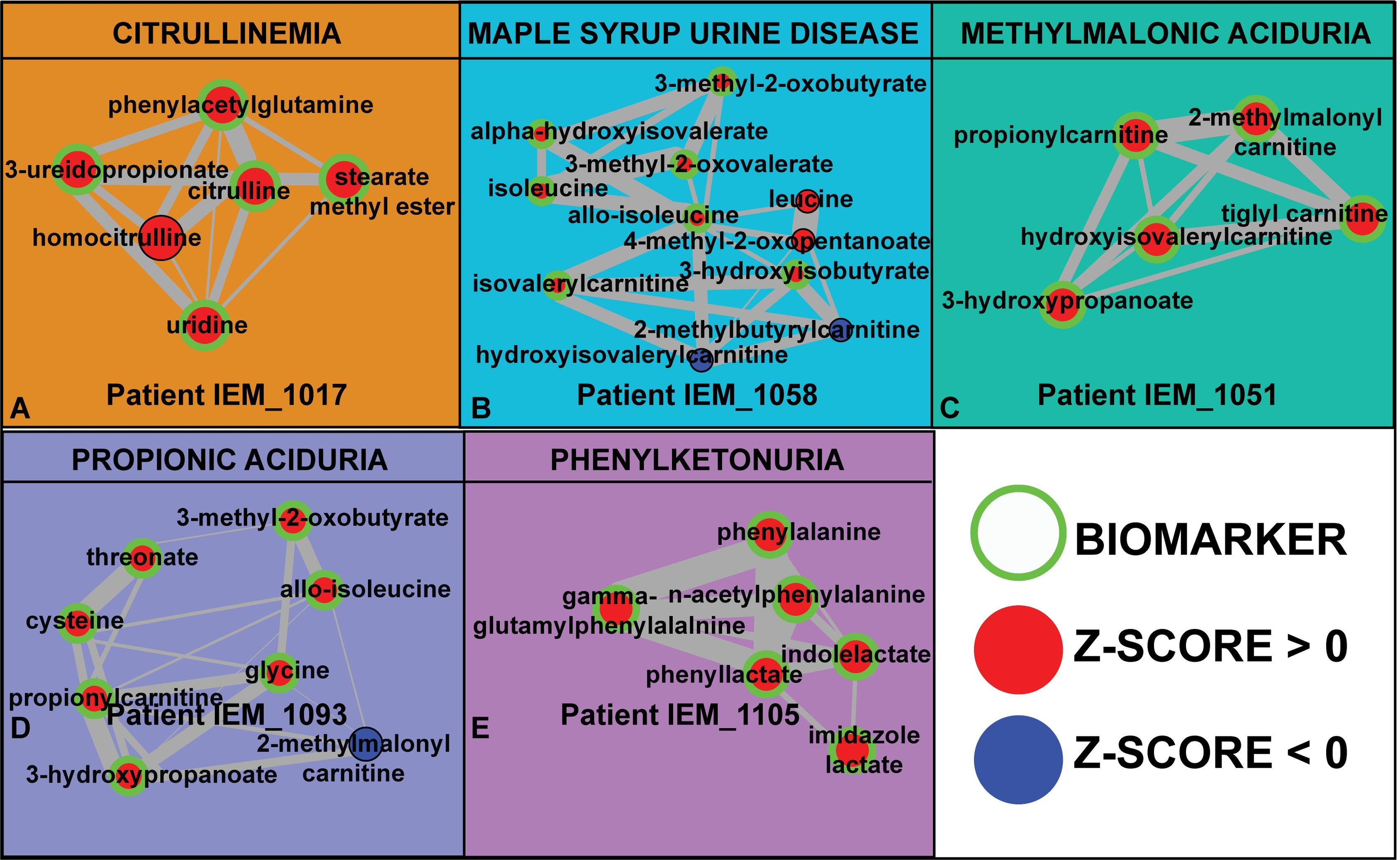

Supplement: S1 Fig — Disease-relevant metabolite features shown in a network context are visualized for select patients from Miller et al. (2015) with known diagnoses. In each of the modules detected for each patient in (A)-(E), several known biomarkers associated with the patient’s true diagnosis are included, indicating that the metabolite features selected by CTD as informative to diagnosis are also clinically relevant for diagnosis. (A) Patient IEM_1017 was diagnosed with citrullinemia, and the module detected for this patient included biomarkers for citrullinemia, such as citrulline, homocitrulline, 3-ureidopropionate and uridine. (B) Patient IEM_1058 was diagnosed with maple syrup urine disease (MSUD), and the module detected for this patient included biomarkers for MSUD such as allo-isoleucine, 4-methyl-2-oxopentanoate, 3-methyl-2-oxobutyrate, leucine, isoleucine, alpha-hydroxyisovalerate, 3-hydroxyisobutyrate, isovalerylcarnitine, 2-methylbutyrylcarnitine, and hydroxyisovalerylcarnitine. (C) Patient IEM_1051 was diagnosed with methylmalonic aciduria (MMA), and the module detected for this patient included biomarkers for MMA, such as 2-methylmalonyl carnitine, propionylcarnitine, tiglyl carnitine, hydroxyisovalerylcarnitine, and 3-hydroxypropanoate. (D) Patient IEM_1093 was diagnosed with propionic aciduria (PA), and the module detected for this patient included biomarkers for PA, such as 2-methylmalonyl carnitine, propionylcarntine, 3-hydroxypropanoate, and glycine. (E) Patient IEM_1105 was diagnosed with phenylketonuria (PKU), and the module detected for this patient included biomarkers for PKU, such as phenylalanine, n-acetylphenylalanine, gamma-glutamylphenylalanine, and phenyllactate. (TIF) [file pcbi.1008550.s001.tif]
